# Role of carbonic anhydrase in acute recovery following renal ischemia reperfusion injury

**DOI:** 10.1371/journal.pone.0220185

**Published:** 2019-08-29

**Authors:** Oskar Nensén, Peter Hansell, Fredrik Palm

**Affiliations:** 1 Department of Medical Cell Biology, Uppsala University, Uppsala, Sweden; 2 Uppsala University Hospital, Uppsala, Sweden; University Medical Center Utrecht, NETHERLANDS

## Abstract

Ischemia reperfusion (IR) injury can cause acute kidney injury. It has previously been reported that kidney oxygen consumption (QO_2_) in relation to glomerular filtration rate (GFR), and thus tubular sodium load, is markedly increased following IR injury, indicating reduced electrolyte transport efficiency. Since proximal tubular sodium reabsorption (TNa) is a major contributor to overall kidney QO_2_, we investigated whether inhibition of proximal tubular sodium transport through carbonic anhydrase (CA) inhibition would improve renal oxygenation following ischemia reperfusion. Anesthetized adult male Sprague Dawley rats were administered the CA inhibitor acetazolamide (50 mg/kg bolus iv), or volume-matched vehicle, and kidney function, hemodynamics and QO_2_ were estimated before and after 45 minutes of unilateral complete warm renal ischemia. CA inhibition *per se* reduced GFR (-20%) and TNa (-22%), while it increased urine flow and urinary sodium excretion (36-fold). Renal blood flow was reduced (-31%) due to increased renal vascular resistance (+37%) without affecting QO_2_. IR *per se* resulted in similar decrease in GFR and TNa, independently of CA activity. However, the QO_2_/TNa ratio following ischemia-reperfusion was profoundly increased in the group receiving CA inhibition, indicating a significant contribution of basal oxygen metabolism to the total kidney QO_2_ following inhibition of proximal tubular function after IR injury. Ischemia increased urinary excretion of kidney injury molecule-1, an effect that was unaffected by CA. In conclusion, this study demonstrates that CA inhibition further impairs renal oxygenation and does not protect tubular function in the acute phase following IR injury. Furthermore, these results indicate a major role of the proximal tubule in the acute recovery from an ischemic insult.

## Introduction

Acute kidney injury (AKI), a sudden decline in renal function, is a common feature in the critically ill patient in the emergency and intensive care setting and is associated with a significant increase in morbidity and mortality [1, 2]. Ischemia reperfusion (IR) injury is one of the most common causes of clinically manifest AKI and seen following major abdominal vascular surgery, cardio-pulmonary bypass, renal transplantation, and hypoperfusion due to circulatory shock [3–7]. Regardless of underlying cause, IR injury is characterized by severely deranged oxygen homeostasis and subsequent loss of cell function. The kidney is especially vulnerable to ischemic injury due to its heterogeneous blood flow. The kidneys receive about 20% of cardiac output under normal physiological conditions, which mainly perfuses the cortex, whereas the renal medulla is functioning on the brink of hypoxia receiving only 10% of total renal blood flow (RBF). This leaves the renal medulla particularly vulnerable due to its high oxygen consumption (QO_2_) required to maintain steep osmotic gradients essential to the kidneys ability to concentrate the urine [8]. Furthermore, since under most conditions increased RBF results in increased GFR and thereby increased tubular sodium load, the kidney cannot improve medullary oxygenation through increased RBF [9]. Thus, therapies that increase GFR while not increasing renal oxygen delivery (RDO_2_) could potentially further impair kidney function and recovery.

Renal QO_2_ is tightly linked to the tubular reabsorption of Na (TNa) along the nephron. The proximal tubule is the primary site of tubular sodium transport, accounting for approximately 60–70% of total TNa. The sodium-hydrogen exchanger 3 (NHE_3_) is the primary sodium transporter in the proximal tubule accounting for a majority of proximal TNa and tubular fluid reabsorption and is dependent on carbonic anhydrase (CA) for the supply of protons [10]. In the proximal tubular cell, CA IV is expressed in the apical membrane while CA II is expressed in the cytosol. Both isoforms are sensitive to inhibition by acetazolamide.

QO_2_ in relation to TNa is increased after IR injury [11] thereby indicating a reduced transport efficiency. It has been speculated that loss of epithelial integrity in the tight junctions and an increased permeability with sodium back flow or disruption in local nitric oxide regulation could account for this dissociation between sodium reabsorption and renal QO_2_ [12, 13]. Since proximal TNa is a major contributor to overall renal QO_2_ we investigated the impact of reduced proximal TNa, achieved by CA inhibition, on kidney function and oxygenation following IR injury to test the hypothesis that proximal tubular function affects the recovery during the acute phase following an ischemic insult to the kidney.

## Material and methods

### Animals

Twenty-three male Sprague Dawley rats (Charles River, Germany) weighing approximately 350 g were used. Rats had access to standard rat chow and water *ad libitum* throughout the duration of the study. All procedures and handling were in accordance with European guidelines for care of laboratory animals and approved by the Animal Care and Use Committee for Uppsala University (C137/15).

### Surgical procedure

Rats were anesthetized by intraperitoneal injection of thiobutabarbital (Inactin, Sigma-Aldrich, St. Louis, MO, USA) 120 mg/kg and placed on a heating pad maintaining core body temperature at 37°C. A tracheostomy was performed to facilitate spontaneous breathing. The left femoral artery was cannulated allowing for measurement of blood pressure and taking arterial blood samples, and the femoral vein for a continuous infusion of a Ringer´s solution containing tritiated inulin ([^3^H]-Inulin 5 ml/kg/h). The bladder was catheterized through a supra pubic incision. The left kidney was accessed through a flank incision and stabilized in a plastic cup and covered in cotton wool soaked in mineral oil to avoid evaporation. The ureter was cannulated allowing for unilateral urine collection and the renal artery separated from the vein and an ultrasound probe (Transonic Systems, NY, USA) was placed around the artery for measurement of total RBF. The renal vein was cannulated with a heparinized silicon catheter with an outer diameter of 1 mm allowing for renal vein blood sampling. Rats were allowed a 45 minute period for recovery from surgery before beginning the experiment. After the conclusion of the protocol the rats were euthanized by an intravenous bolus injection of saturated potassium chloride.

### Experimental protocol

Rats were assigned as vehicle treated controls (n = 12) or treated with 50 mg/kg i.v acetazolamide (Diamox, Wyeth Lederle S.p.A., Catania, Italy, 100 mg/ml; n = 11) after the 45 minute recovery period. A 40 minute baseline collection period was started 5 minutes following administration of vehicle/acetazolamide after which 45 minutes of warm ischemia was induced by clamping of the renal pedicle. Following ischemia, a 2 h reperfusion period was conducted after which a second 40 minute collection period was carried out. An arterial blood sample was obtained in the middle of each collection period for calculation of GFR. Arterial and renal vein samples were obtained at the end of each collection period and blood gases measured using the iSTAT system (Abbott Laboratories, IL, USA). Arterial blood pressure and total RBF were measured continuously throughout the experiment.

### Excretory parameters and calculations

Urine flow was measured gravimetrically. GFR was calculated using inulin clearance. Urinary and plasma ^3^H activities were determined by liquid scintillation spectrometry (Tri-Carb 2910 TR, PerkinElmer, Waltham, MA, USA). Plasma and urinary electrolyte concentrations were determined using flame photometry (model IL543, Instrumentation Lab, Milan, Italy). Urinary KIM-1 was determined by ELISA (R&D Systems Europe, Abingdon, UK) according to the manufacturers’ instructions.

GFR was calculated as C_u_*U_v_/C_p;_ where C_u_ = urinary ^3^H activity, U_v_ = urine flow (μl/min) and C_p_ = plasma ^3^H activity. Arterial and venous oxygen content was calculated using the standard formula: Hb*1.34*(SO_2_/100)+(PO_2_*0.0022) where Hb = hemoglobin concentration (g/l), SO_2_ = hemoglobin saturation, and PO_2_ partial pressure of oxygen. QO_2_ was estimated by the arteriovenous difference in oxygen content multiplied by RBF. RDO_2_ was calculated by arterial oxygen content multiplied by RBF. Renal oxygen extraction was calculated as QO_2_ divided by RDO_2_.

Tubular sodium transport (TNa) was calculated as [P_Na_]*GFR-[U_Na_]*U_v_, where [P_Na_] = plasma sodium concentration and [U_Na_] = urinary sodium concentration. Fractional sodium excretion (FE_Na_) was given by sodium clearance/GFR*100, where sodium clearance = ([U_Na_]* U_v_)/[P_Na_].

KIM-1 was normalized to GFR, calculated as KIM-1 excretion per minute divided by GFR giving KIM-1 excreted per volume filtrate.

### Statistics

Data were analyzed using repeated measures two-way ANOVA with Fisher’s LSD post-hoc test (Prism 7, GraphPad Software, La Jolla, CA, USA). P<0.05 was considered statistically significant. All data are presented as mean±SEM.

## Results

**Fig 1:**
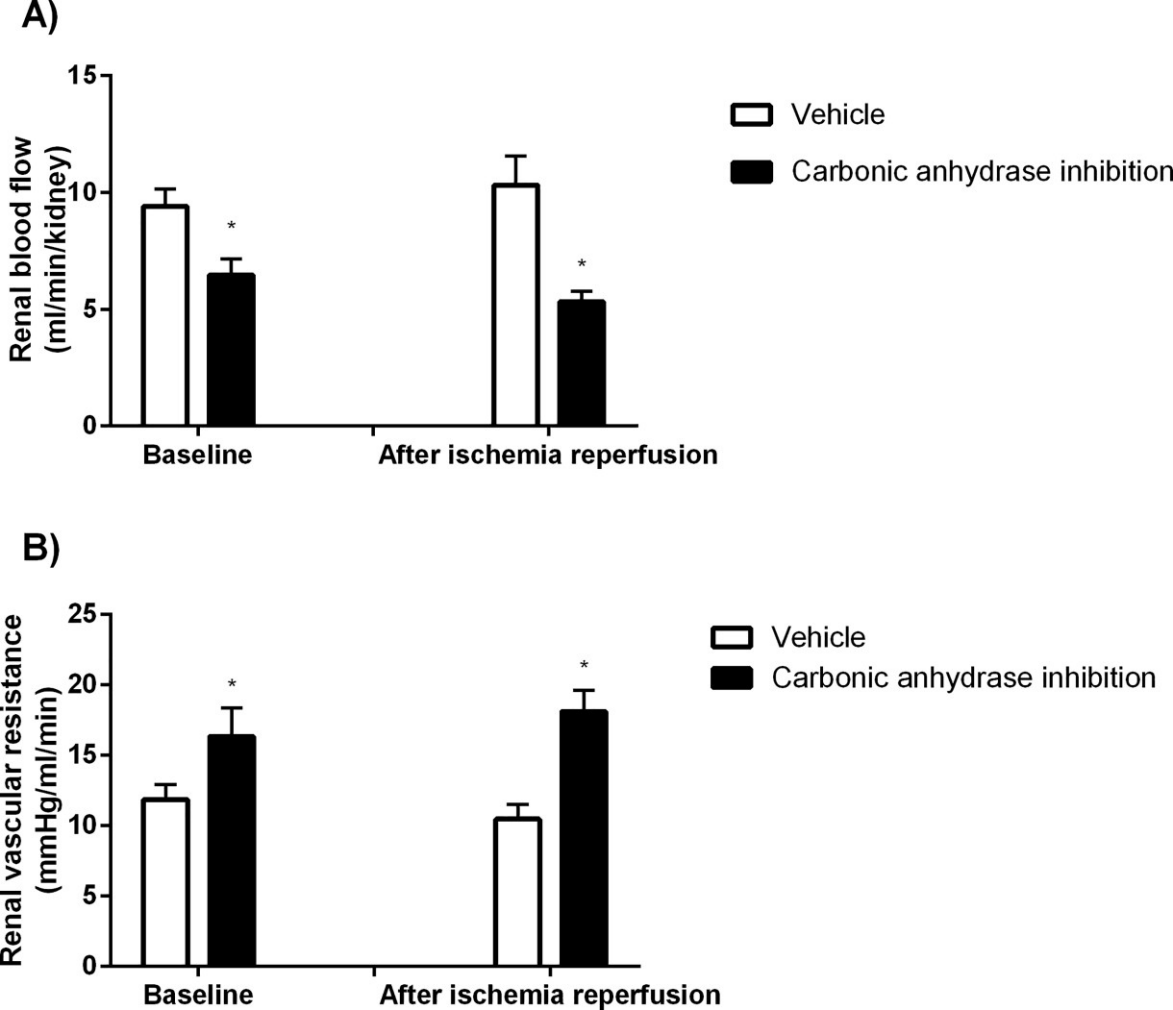
Renal hemodynamics. Renal blood flow (A) and renal vascular resistance (B) at baseline and following ischemia reperfusion after CA inhibition (n = 11) and corresponding vehicle treatment (n = 12). * denotes P<0.05 vs corresponding vehicle.

CA inhibition induced systemic acidosis, decreased base excess (Table 1) and reduced mean arterial blood pressure (104±2 vs. 93±3 mmHg; P<0.05). RBF was also reduced in response to CA inhibition due to increased RVR (Fig 1).

**Table 1:** Arterial blood chemistry.

Group		pH	pCO_2_ (kPa)	pO_2_ (kPa)	BE (mmol/l)	[HCO_3_] (mmol/l)	TCO_2_ (mmol/l)	sO_2_ (%)	[Na^+^] (mmol/l)	[K^+^] (mmol/l)	[Hb] (g/l)
Vehicle	Baseline	7.34±0.01	7.7±0.3	9.2±0.3	5.8±0.7	31.5±0.7	33.3±0.8	91±1	139±1	4.6±0.1	139±3
	After IR	7.38±0.01†	6.6±0.3†	9.5±0.4	3.7±0.8†	28.9±0.8†	30.4±0.8†	93±1†	140±1	4.1±0.1†	133±3
Carbonic anhydrase inhibition	Baseline	7.17±0.01*	10.3±.0.3*	10.6±0.5*	-0.2±0.5*	28.3±0.5*	30.6±0.6*	90±2	140±0	3.7±0.1*	148±2
	After IR	7.15±0.01*†	8.4±0.5*†	12.1±0.5*†	-7.6±6.6*†	21.5±0.6*†	23.2±0.7*†	93±1††	138±1*†	4.9±0.2*†	126±6†
***ANOVA***											
Group		P<0.05	P<0.05	P<0.05	P<0.05	P<0.05	P<0.05	ns	ns	ns	ns
Time		ns	P<0.05	P<0.05	P<0.05	P<0.05	P<0.05	P<0.05	ns	P<0.05	P<0.05
Interaction		P<0.05	P<0.05	P<0.05	P<0.05	P<0.05	P<0.05	ns	P<0.05	P<0.05	P<0.05

pCO_2_ –partial pressure of carbon dioxide; pO_2_ –partial pressure of oxygen; BE–base excess; TCO_2_ –total carbon dioxide; sO_2_ –oxygen saturation; HB–hemoglobin.

* denotes P<0.05 vs corresponding vehicle

† denotes P<0.05 vs baseline within same group.

ns = not statistically significant. Values are mean ±SEM.

**Fig 2:**
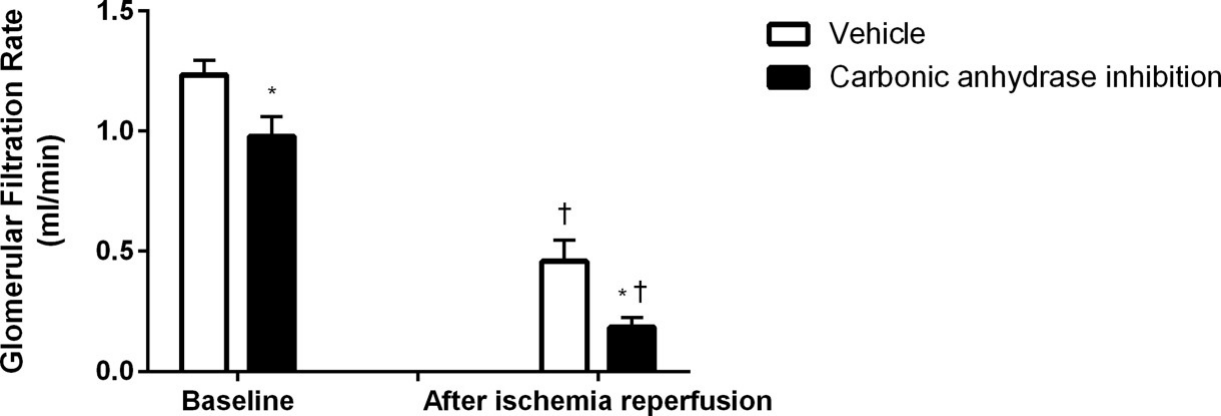
Glomerular filtration rate. Glomerular filtration rate at baseline and following ischemia reperfusion after CA inhibition (n = 11) and corresponding vehicle treatment (n = 12). * denotes P<0.05 vs corresponding vehicle, † denotes P<0.05 vs baseline within same group.

**Fig 3:**
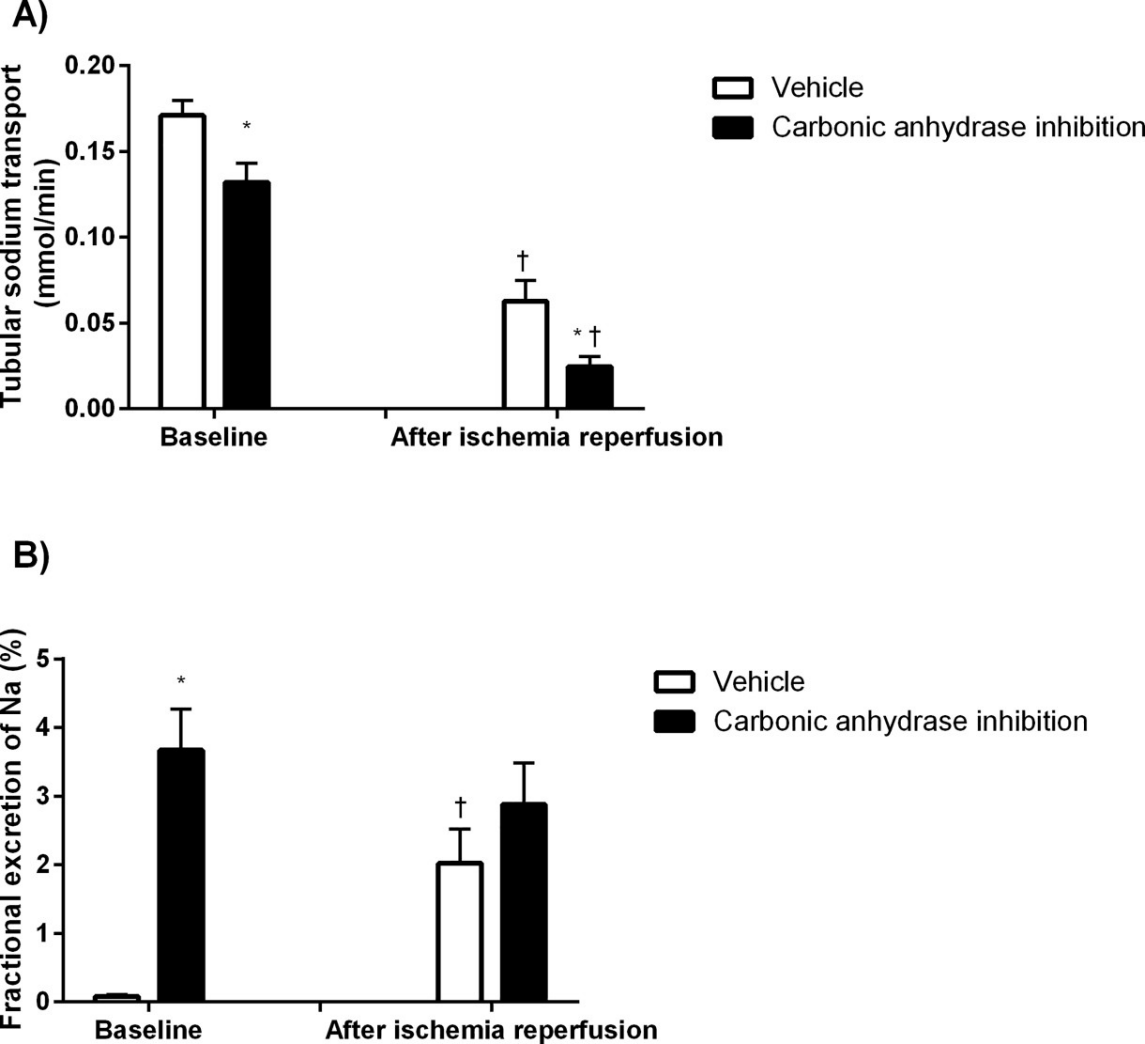
Tubular sodium handling. Tubular sodium transport (A) and fractional excretion of sodium (B) at baseline and following ischemia reperfusion after CA inhibition and corresponding vehicle treatment (n = 12). * denotes P<0.05 vs corresponding vehicle, † denotes P<0.05 vs baseline within same group.

CA inhibition *per se* reduced GFR (Fig 2) and TNa (Fig 3A) whereas it increased FENa (Fig 3B). Urine flow was increased at baseline by CA inhibition (3.5±0.5 vs 37.2±2.7 μl/min; P<0.05 for control and treated animas respectively) while following IR, CA inhibition reduced urine flow rate (11.2±1.3 vs 6.7±1.6 μl/min; P<0.05 for control and treated animals, respectively).

**Fig 4:**
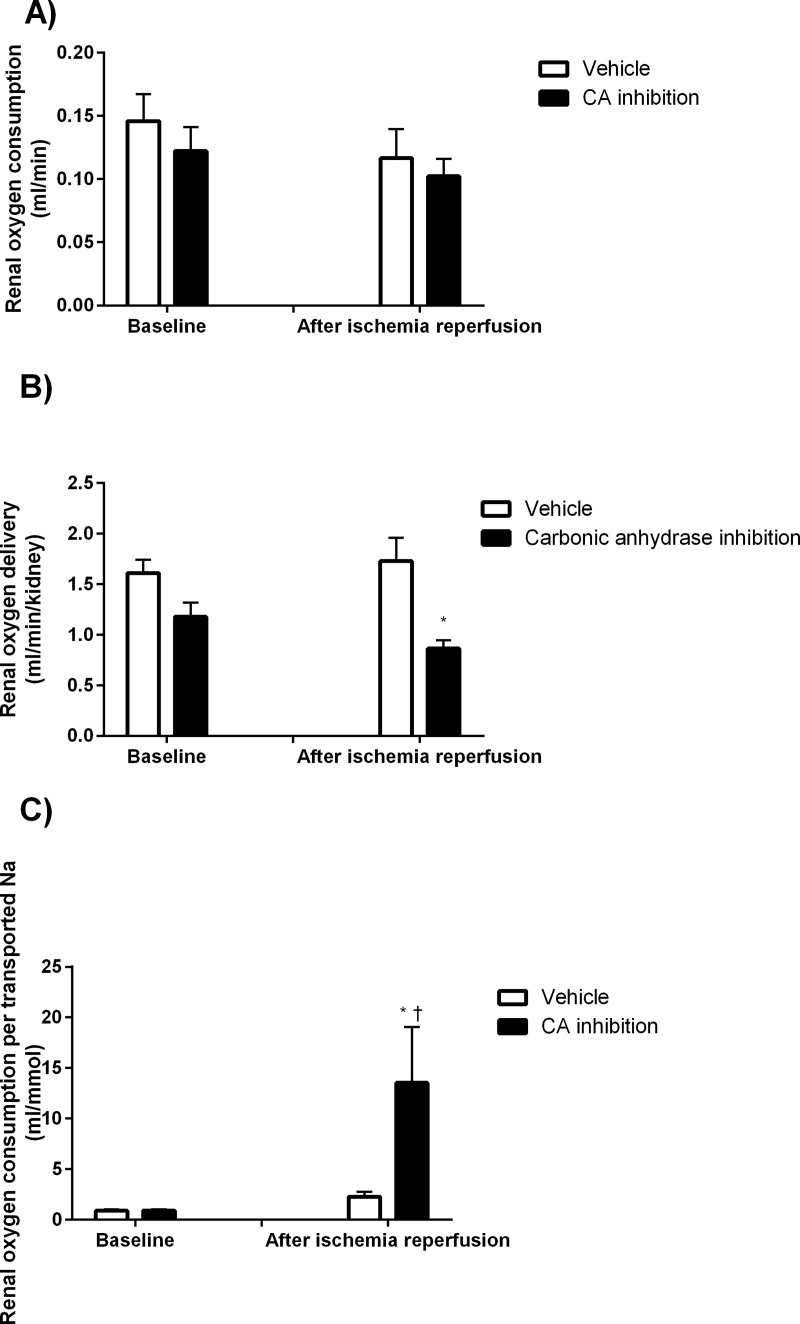
Renal oxygenation. Renal oxygen consumption (A), renal oxygen delivery (B) and tubular sodium transport efficiency (C) at baseline and following ischemia reperfusion after CA inhibition (n = 11) and corresponding vehicle treatment (n = 12).* denotes P<0.05 vs corresponding vehicle, † denotes P<0.05 vs baseline within same group.

However, CA inhibition did not affect total kidney QO_2_ (Fig 4A), renal oxygen delivery (Fig 4B) or oxygen extraction (10±1% vs 12 ±2%; ns for control and treated animals, respectively). Following IR however, CA inhibition increased oxygen extraction (7±1% vs 12±1%; P<0.05 for control and treated animals, respectively).

There was a similar decrease in relation to baseline in GFR (-60±9 vs -79±5%; ns, Fig 2) and TNa (Fig 3A) in both groups in response to IR. QO_2_ was maintained in both groups following IR (Fig 4A). CA inhibition significantly increased QO_2_/TNa following IR (Fig 4C).

Animals subjected to CA inhibition maintained reduced RBF and increased RVR following IR vs controls (Fig 1). MAP was reduced in the control group following IR (104±2 vs 96±4 mmHg; P<0.05) while in the treatment group there was no significant decrease vs baseline values (93±3 vs 91±4 mmHg; ns), and there was no significant difference in MAP between groups following IR.

**Fig 5:**
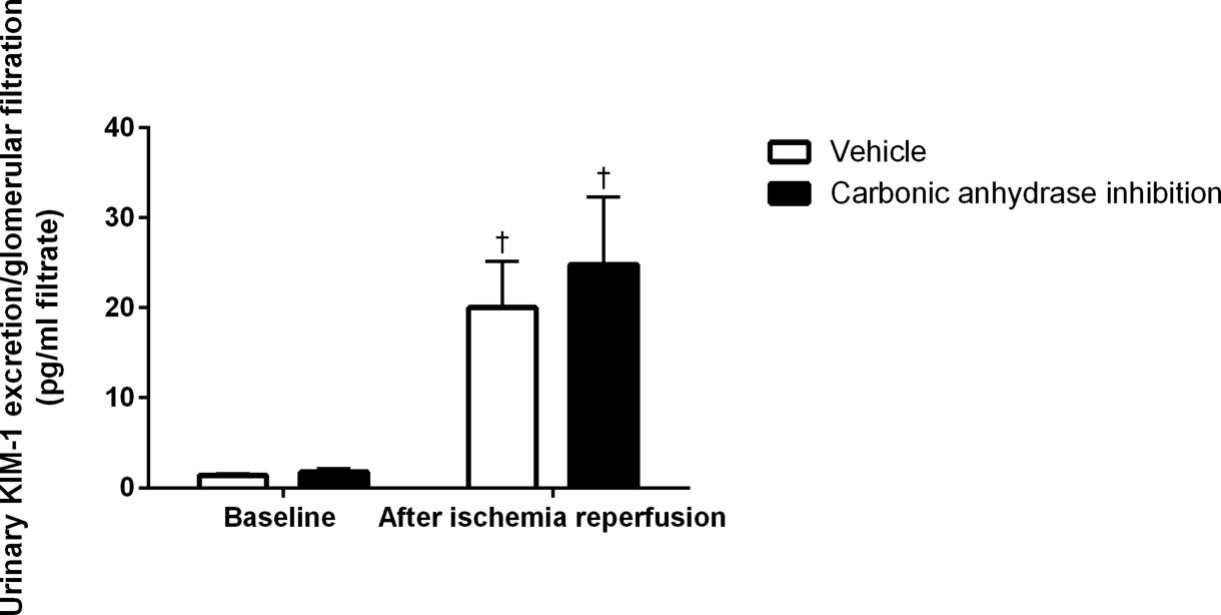
KIM-1. Urinary excretion of the kidney injury molecule (KIM)-1 at baseline and following ischemia reperfusion after CA inhibition (n = 9) and corresponding vehicle treatment (n = 12). † denotes P<0.05 vs baseline within same group.

Urinary KIM-1 excretion was not affected by CA-inhibition prior to IR. IR caused a similar increase in urinary KIM-1 excretion in both vehicle and CA-inhibitor treated animals (Fig 5). KIM-1 could not be analyzed in one animal receiving CA inhibition due to technical issues.

## Discussion

In the present study, we demonstrate that renal QO_2_/TNa is significantly increased in the acute phase after IR when inhibiting proximal TNa, which indicates reduced transport efficiency. This occurs despite decreased RDO_2_ which potentially further impairs renal oxygenation via exaggerated mismatch in the renal oxygen supply/demand relationship. These results demonstrate the pivotal role of maintained proximal tubular function, and CA activity, in the acute recovery following IR injury.

Under normal physiological conditions there is an almost perfect linear relationship between TNa and QO_2_ [9]. However, following IR injury renal QO_2_ in relation to GFR, and thus tubular sodium load, is markedly increased, indicative of less efficient TNa. Additionally, the increased QO_2_ is not compensated by a corresponding increase in RDO_2_, potentially exacerbating renal hypoxia [11]. It has previously been demonstrated that the use of loop diuretics, to inhibit NKCC2 activity, improves medullary oxygenation following acute IR injury [14]; and while CA inhibition has been demonstrated to increase cortical oxygen tension (PO_2_) under normal conditions [15], its role in proximal tubular function has been left largely unexplored. In the present study, we used a model of a moderately severe unilateral warm ischemia in order to achieve a 60–70% reduction in GFR. We observed similar decreases in relation to baseline GFR in both groups, while absolute values were lower in the animals receiving CA inhibition. The inhibition of CA had no statistically significant effect on total QO_2_ at either baseline or following IR in accordance with previous findings [16]. Since QO_2_ was equal in both groups despite GFR being markedly lower after CA inhibition, this resulted in increased QO_2_/TNa indicative of less efficient TNa.

The severity of renal injury following IR is proportional to the duration of ischemia [17]. Increasing duration of ischemia before restoration of blood flow results in edema and swelling of the tissue, which compromises reperfusion and elevates biomarkers of kidney injury [17, 18]. It is possible that the limited ischemia duration did not cause kidney function in the control group to decline to the extent where it would be reflected in the QO_2_/TNa in this group.

KIM-1 is expressed in renal tubular cells at low levels in the normal kidney and is dramatically upregulated following renal injury, and as such, may be used as a marker of renal injury [19, 20]. Indeed, urinary KIM-1 excretion per volume filtrate following IR was increased in all animals and CA inhibition had no effect. It is however possible that we are too early to detect differences in-between groups.

In isolated mitochondria from transplanted kidneys, uncoupling of the electron transport chain is evident as demonstrated by increased mitochondrial QO_2_ unrelated to ATP production [21]. This might in part account for the lack of a proportional decrease in QO_2_ to that of GFR. Additionally, inhibition of proximal TNa shifts TNa to more distal parts of the nephron which are reported to require more QO_2_ in order to reabsorb the same amount of Na [22].

CA inhibition reduced RBF due to increased renal vascular resistance. It has previously been demonstrated that CA inhibition reduces RBF via activation of the tubuloglomerular feedback mechanism due to the increased Na load to the macula densa [23]. This reduction in RBF resulted in a reduced RDO_2_ despite maintained QO_2_ following IR, i.e. a shift in the oxygen supply/demand relationship. While CA inhibition had no significant effects on RDO_2_or extraction at baseline, following IR RDO_2_ was reduced compared to controls while oxygen extraction was increased. This is suggestive of CA being important in the acute recovery following IR injury by influencing renal oxygen homeostasis. Additionally, through activation of tubuloglomerular feedback as well as increased proximal tubular pressure, CA inhibition decreases GFR.

The findings of the present study where QO_2_/TNa was increased following inhibition of proximal TNa suggests that, at least in this tubular segment, epithelial leakage and thus futile TNa is not the main causes of increased QO_2_/TNa. Rather, the increase in QO_2_/TNa suggests that maintaining proximal tubular function is critical in maintaining renal oxygen homeostasis and that the inhibition of TNa in the proximal tubule in shifting TNa to more distal parts of the nephron exacerbates the oxygen supply/demand mismatch.

CA inhibition resulted in acidosis at baseline, which remained constant throughout the experiment. Tight control of metabolic acidosis following renal transplantation has been shown to reduce serum creatinine levels during the first week following transplantation suggesting that acidosis contributes to kidney injury in this patient population [24]. In this experimental approach, we cannot exclude the possibility of acidosis as a contributing factor to the increased susceptibility to IR injury despite acidosis *per se* having no impact on kidney function at baseline. Additionally, rates were kept under general anesthesia throughout the experiment, which may influence the effects of acetazolamide.

## Conclusions

Reduced proximal TNa makes the kidneys more vulnerable to IR injury by altering the renal oxygen supply/demand relationship. Furthermore, inhibiting active TNa in the proximal tubule further deteriorates renal function in the acute recovery following an ischemic event. This indicates a major role of the proximal tubule in maintaining kidney function and oxygen homeostasis in the acute recovery following IR injury.

## Supporting information

S1 Minimal data set Minimal data set. The minimal data set for all figures included in the paper.
(DOCX)
