# Supplementary material for: Role of carbonic anhydrase in acute recovery following renal ischemia reperfusion injury
Source: PLoS One. 2019 Aug 29;14(8):e0220185. doi: 10.1371/journal.pone.0220185 (PMC6715224; doi:10.1371/journal.pone.0220185)
Supplement: S1 Minimal data set — The minimal data set for all figures included in the paper. (DOCX) [file pone.0220185.s001.docx]

**Supporting information**

Dataset used for calculating the means and standard deviations for each figure.

| Fig. # | Mean | S.D. | S.E.M. | Statistical methods used | P value | # samples |
| --- | --- | --- | --- | --- | --- | --- |
| **Fig. 1A** |  |  |  | Repeated measures two-way ANOVA and Fisher´s LSD | *P<0.05 |  |
| Vehicle baseline | 9.42 | 2.56 | 0.74 |  |  | 12 |
| CAI baseline | 6.46 | 2.35 | 0.71 |  |  | 11 |
| Vehicle after IRI | 10.31 | 4.32 | 1.25 |  |  | 12 |
| CAI after IRI | 5.34 | 1.45 | 0.44 |  |  | 11 |
|  |  |  |  |  |  |  |
| **Fig. 1B** |  |  |  | Repeated measures two-way ANOVA and Fisher´s LSD | *P<0.05 |  |
| Vehicle baseline | 11.87 | 3.61 | 1.04 |  |  | 12 |
| CAI baseline | 16.34 | 6.69 | 2.02 |  |  | 11 |
| Vehicle after IRI | 10.47 | 3.62 | 1.05 |  |  | 12 |
| CAI after IRI | 18.12 | 4.97 | 1.50 |  |  | 11 |
|  |  |  |  |  |  |  |
| **Fig. 2** |  |  |  | Repeated measures two-way ANOVA and Fisher´s LSD | *P<0.05  †P<0.05 |  |
| Vehicle baseline | 1.23 | 0.21 | 0.06 |  |  | 12 |
| CAI baseline | 0.98 | 0.27 | 0.08 |  |  | 11 |
| Vehicle after IRI | 0.46 | 0.31 | 0.09 |  |  | 12 |
| CAI after IRI | 0.18 | 0.14 | 0.04 |  |  | 11 |
|  |  |  |  |  |  |  |
| **Fig. 3A** |  |  |  | Repeated measures two-way ANOVA and Fisher´s LSD | *P<0.05  †P<0.05 |  |
| Vehicle baseline | 0.17 | 0.03 | 0.009 |  |  | 12 |
| CAI baseline | 0.13 | 0.04 | 0.011 |  |  | 11 |
| Vehicle after IRI | 0.06 | 0.04 | 0.012 |  |  | 12 |
| CAI after IRI | 0.02 | 0.02 | 0.006 |  |  | 11 |
|  |  |  |  |  |  |  |
| **Fig. 3B** |  |  |  | Repeated measures two-way ANOVA and Fisher´s LSD | *P<0.05  †P<0.05 |  |
| Vehicle baseline | 0.08 | 0.08 | 0.02 |  |  | 12 |
| CAI baseline | 3.67 | 1.97 | 0.59 |  |  | 11 |
| Vehicle after IRI | 2.02 | 1.72 | 0.50 |  |  | 12 |
| CAI after IRI | 2.88 | 1.99 | 0.60 |  |  | 11 |
|  |  |  |  |  |  |  |
| **Fig. 4A** |  |  |  | Repeated measures two-way ANOVA and Fisher´s LSD |  |  |
| Vehicle baseline | 0.15 | 0.07 | 0.02 |  |  | 12 |
| CAI baseline | 0.12 | 0.06 | 0.02 |  |  | 11 |
| Vehicle after IRI | 0.11 | 0.08 | 0.02 |  |  | 12 |
| CAI after IRI | 0.10 | 0.05 | 0.01 |  |  | 11 |
|  |  |  |  |  |  |  |
| **Fig. 4B** |  |  |  | Repeated measures two-way ANOVA and Fisher´s LSD | *P<0.05 |  |
| Vehicle baseline | 1.61 | 0.45 | 0.13 |  |  | 12 |
| CAI baseline | 1.18 | 0.46 | 0.14 |  |  | 11 |
| Vehicle after IRI | 1.73 | 0.78 | 0.23 |  |  | 12 |
| CAI after IRI | 0.86 | 0.28 | 0.08 |  |  | 11 |
|  |  |  |  |  |  |  |
| **Fig. 4C** |  |  |  | Repeated measures two-way ANOVA and Fisher´s LSD | *P<0.05  †P<0.05 |  |
| Vehicle baseline | 0.88 | 0.50 | 0.14 |  |  | 12 |
| CAI baseline | 0.91 | 0.39 | 0.12 |  |  | 11 |
| Vehicle after IRI | 2.25 | 1.75 | 0.51 |  |  | 12 |
| CAI after IRI | 13.54 | 18.36 | 5.54 |  |  | 11 |
|  |  |  |  |  |  |  |
| **Fig. 5** |  |  |  | Repeated measures two-way ANOVA and Fisher´s LSD | †P<0.05 |  |
| Vehicle baseline | 1.40 | 0.48 | 0.14 |  |  | 12 |
| CAI baseline | 1.76 | 1.04 | 0.35 |  |  | 9 |
| Vehicle after IRI | 20.00 | 17.88 | 5.16 |  |  | 12 |
| CAI after IRI | 24.83 | 22.45 | 7.49 |  |  | 9 |

CAI - carbonic anhydrase inhibition; IRI – ischemia reperfusion injury
